# Impact of pre-COVID-19 epidemic preparedness on the trajectory of the pandemic in African countries

**DOI:** 10.4102/ajlm.v11i1.1571

**Published:** 2022-03-31

**Authors:** Talkmore Maruta, Sikhulile Moyo

**Affiliations:** 1 Laboratory Department, African Centres for Disease Control and Prevention, Lusaka, Zambia; 2 Laboratory Department, Botswana Harvard AIDS Institute Partnership, Gaborone, Botswana

**Keywords:** COVID-19, preparedness, response, World Health Organization Joint External Evaluation, Global Health Security Index

## Abstract

**Background:**

The novel coronavirus disease 2019 (COVID-19), declared a pandemic by the World Health Organization (WHO) in March 2020, has taught us about the importance of epidemic preparedness.

**Objective:**

We analysed the pre-COVID-19 preparedness of sub-Saharan African countries and how this may have influenced the trajectory of COVID-19 cases.

**Methods:**

The WHO Joint External Evaluation (JEE) tool and the Global Health Security (GHS) Index were used to determine the epidemic preparedness of countries in the WHO African Region. The relationship between pre-COVID-19 preparedness and the reported number of cases per million people was evaluated over the first 120 days of the first reported case in each country, between February 2020 and September 2020.

**Results:**

The overall performance of the 42 countries was 40% in the 19 JEE core capacities and 32% in the six GHS Index indicators. At Day 1, the mean number of cases per million population was significantly higher among countries rated as ‘prepared’ in the JEE legislation, policy and finance (*p* = 0.03), ports of entry (*p* = 0.001), and international health regulation coordination, communication and advocacy (*p* = 0.03) categories. At Day 90, countries rated as ‘prepared’ in the national laboratory systems (*p* = 0.05) and real-time surveillance (*p* = 0.04) JEE categories had statistically significantly fewer cases per million population.

**Conclusion:**

This analysis highlights the importance of building capacity for pandemic preparedness in Africa. The WHO African Region was not adequately prepared for the COVID-19 pandemic as measured by the WHO JEE tool and the GHS Index.

## Introduction

The rapid spread of the novel coronavirus disease 2019 (COVID-19) prompted the World Health Organization (WHO) to declare it as a public health emergency of international concern in January 2020 and a global pandemic on 11 March 2020.^1,2^ The first COVID-19 case in Africa was reported by Egypt on 14 February, and by 14 May, all African countries had reported at least one COVID-19 case.^3^ By June 2020, 152 442 COVID-19 cases and 4334 deaths (case fatality rate: 3%) had been reported by the 54 African countries.^4^ African countries instituted various public health and social measures to curb the transmission and allow them to prepare for the pandemic.^5^

Although the coronavirus was novel, the emergency of its spread exposed the status of countries’ preparedness for threats posed by epidemics, disasters, and other events of public health concern. Countries are expected to invest resources to limit the impact of disease outbreaks and natural disasters.^6^ Epidemic preparedness is a measure of the capacity of countries to detect, report, and respond to outbreaks. To effectively respond to outbreaks, countries need to set up robust systems for surveillance and outbreak investigation with capabilities to rapidly identify, characterise, and track emerging infectious diseases.^6^ For a coordinated response, these systems need to function within strong national public health systems linked to an effective international system.^6^

The 2014 Ebola outbreak in West Africa infected 28 000 and killed 10 000 people in the three affected countries of Guinea, Liberia, and Sierra Leone, re-emphasising that epidemics can emerge unexpectedly and unprepared countries are a threat to all.^7^ The estimated combined loss was $2.8 billion in gross domestic product.^7^ This outbreak prompted globally coordinated efforts to ensure that countries strengthen their capacities as a way of ensuring that threats are limited and contained within borders when they occur.

The International Health Regulations of 2005 (IHR 2005), among other innovations, was a means to ensure that countries are obliged to develop minimum capacities, defined as core public health capacities.^8^ Due to the limited implementation of IHR 2005, especially in Africa, the WHO developed the Integrated Disease Surveillance and Response (IDSR) to improve surveillance by linking community, health facility, district, and national levels in a streamlined manner using a One Health Approach.^9^ However, by the 2012 deadline, only 58% of the signatories had developed national plans to meet IHR 2005 core capacity requirements, with as few as 10% indicating full implementation of the requirements. Further to that, the Global Health Security Agenda was launched in 2014 to address the limited implementation of global commitments to building capacities for preparedness for epidemics and other events of public health concern using a multisectoral approach that includes the human, agriculture, animal, security, finance, border control, education, and research sectors.^10^

The impact of epidemics is well understood by governments.^11^ In addition to loss of lives, the cost to the broader economic and social sectors is a notable impact of epidemics, with epidemic response costlier than investing in preparedness. Despite all this, the argument to invest in preparedness has not been won by many governments. Ten years after the Abuja Declaration, where leaders of African Union countries pledged to allocate at least 15% of their annual budget to the health sector, only 26 countries had increased the proportion of total government expenditures for health, with only one, Tanzania, having achieved the 15% target.^11^ Within that health budget, pandemic preparedness is often overlooked in favour of more immediate and visible curative demands.^12^

The COVID-19 pandemic once again tested the epidemic preparedness of countries and the global community. In this report, we review the pre-COVID-19 epidemic preparedness of countries in the WHO African Region and its impact on how the virus spread, as well as the strength and effectiveness of the initial responses by countries and the global community. Lessons learnt can be useful for the inevitable future pandemics.

## Methods

### Ethical considerations

The study did not involve human or animal subjects and therefore no ethical approval or clearance was required.

### Study population

Data from the WHO Joint External Evaluations (JEE) conducted between 2016 and 2019 and the Global Health Security (GHS) Index conducted in 2019 were used to determine the levels of preparedness of countries in the WHO African Region. Forty-two countries from the WHO African Region with complete scores for both the WHO JEE and GHS Index were included in the analysis. Data on the reported number of cases per million people from Day 1 (date of the first reported case; range: 28 February 2020 – 15 May 2020) to Day 120 (120 days from the date of the first reported case; range: 26 June 2020 – 11 September 2020) in each of the 42 countries was also obtained and analysed.

### Preparedness data sources

#### World Health Organization JEE

The WHO JEE is a process established by the WHO to assess countries’ capacities to prevent, detect, and rapidly respond to public health risks.^13^ The JEE has two levels of assessment: an initial self-evaluation by the host country using local experts from all relevant sectors and an in-country evaluation conducted by an external team made up of multisectorial subject matter experts and peer countries. The process measures country-specific preparedness for events of public health concern across 19 technical areas, as well as progress made towards achieving IHR 2005 targets.^13^

The scored JEE tool contains a set of questions to collect data on the status of implementation of the 19 technical areas, which are divided into 4 areas, namely prevent, detect, respond, and other IHR-related hazards (Table 1).^14^ Each question is scored from 1, indicating no evidence of implementation, to 5, indicating full implementation.

**TABLE 1:** Sections and technical areas of the WHO JEE tool.

Section	Technical area	Number of questions	Total score	
			*n*	*%*
Prevention	National legislation, policy and financing	2	10	-
	IHR coordination, communication and advocacy	1	5	-
	Antimicrobial resistance	4	20	-
	Zoonotic disease	3	15	-
	Food safety	1	5	-
	Biosafety and biosecurity	2	5	-
	Immunisation	2	10	-
Section total			70	30
Detection	National laboratory system	4	20	-
	Real-time surveillance	4	20	-
	Reporting	2	10	-
	Workforce development	3	15	-
Section total			65	28
Response	Preparedness	2	10	-
	Emergency response operations	4	20	-
	Linking public health and security authorities	1	5	-
	Medical countermeasures and personal deployment	2	10	-
	Risk communication	5	25	-
Section total			70	30
Other IHR-related hazards and points of entry	Points of entry	2	10	-
	Chemical events	2	10	-
	Radiation emergencies	2	10	-
Section total			30	13

*Source:* World Health Organization. Joint external evaluation tool and process overview [homepage on the Internet]. [cited 2020 Jul 2]. Available from: https://apps.who.int/iris/bitstream/handle/10665/252755/WHO-HSE-GCR-2016.18-eng.pdf?sequence=1

IHR, International Health Regulations.

#### Global Health Security Index

The GHS Index is a project of the Nuclear Threat Initiative and the Johns Hopkins Center for Health Security that provides a recurring measure of the international capability for preventing, detecting, and rapidly responding to epidemic and pandemic threats.^15^ The GHS Index tracks health security and related capabilities of the 195 countries that are signatories to the IHR 2005 guidelines and uses a framework with 140 questions organised into six categories, 34 indicators, and 85 sub-indicators (Table 2). The GHS Index was considered as an additional measure of countries’ preparedness as it also includes an assessment of the robustness of the broader healthcare system, national political and socio-economic risks, and adherence to international norms.

**TABLE 2:** Categories, indicators, and sub-indicators of the GHS Index.

Category	Indicator	Number of sub-indicators	Weight (%)
Prevention	Antimicrobial resistance	2	16.1
	Zoonotic disease	5	17.8
	Biosecurity	5	16.1
	Biosafety	2	16.1
	Dual-use research and culture of responsible science	2	14.4
	Immunisation	1	19.5
Total			100.0
Detection	Laboratory systems	3	26.1
	Real-time surveillance and reporting	5	16.9
	Epidemiology workforce	2	25.4
	Data integration between human, animal, and environmental health sectors	1	21.6
Total			100.0
Response	Emergency preparedness and response planning	2	15.7
	Exercising response plans	1	13.7
	Emergency response operation	1	16.8
	Linking public health and security authorities	1	17.7
	Risk communication	2	17.8
	Access to communications infrastructure	4	12.2
	Trade and travel restrictions	2	11.2
Total			100.0
Health Systems	Health capacity in clinics, hospitals, and community care centres	2	17.3
	Medical countermeasures and personnel deployment	3	16.8
	Healthcare access	2	18.4
	Communication with healthcare workers during a public health emergency	1	16.8
	Infection control practices and availability of equipment	2	18.4
	Capacity to test and approve new medical countermeasures	2	12.4
Total			100.0
Commitment	IHR reporting compliance and disaster risk reduction	2	17.4
	Cross-border agreements on public health and animal health emergency response	1	15.7
	International commitments	2	13.5
	JEE and Performance of Veterinary Services Pathway	2	16.3
	Financing	3	19.7
	Commitment to sharing of genetic and biological data and specimens	1	17.4
Total			100.0
Risk and Vulnerability	Political and security risk	7	22.2
	Socio-economic resilience	5	19.0
	Infrastructure adequacy	3	20.3
	Environmental risks	3	17.6
	Public health vulnerabilities	3	20.9
Total			100.0

*Source:* Global Health Security Index. 2019. Building collective action and accountability [homepage on the Internet]. [cited 2020 Jul 2]. Available from: https://www.ghsindex.org/

GHS, Global Health Security; JEE, Joint External Evaluation; IHR, International Health Regulations.

Each country is assigned an overall score between 0% and 100% as a weighted sum of the six categories. To allow for comparisons, each category is normalised based on the sums of its indicators and sub-indicators.

#### The ‘Our World in Data’ programme

The ‘Our World in Data’ is a collaborative programme between the University of Oxford and the Global Change Data Laboratory that, among others, has been collecting data on several areas related to the progress of the COVID-19 pandemic across the globe.^16^ Data on the number of daily confirmed COVID-19 cases per million people from Day 1 (date of the first reported case in each country) to Day 120 (120 days from the date of the first reported case in each country) of the epidemic was collected from the Our World in Data database. Countries’ responses during the first 120 days of the pandemic were taken as a reflection of their existing pre-COVID-19 capacities and preparedness.

The stringency index, which is tracked by ‘Our World in Data’, is also reported. The stringency index is a composite measure calculated from nine response indicators, including school closures, workplace closures, cancellation of public events, restrictions on public gatherings, closures of public transport, stay-at-home requirements, public information campaigns, restrictions on internal movements, and international travel controls and travel bans. Strictness is measured on a scale of 0–100, with 100 being the strictest.

### Determination of preparedness

The determination of pre-COVID-19 preparedness was based on the WHO JEE and GHS indices. The WHO JEE evaluations were conducted between 2016 and 2019. The total scores from each of the 19 technical areas assessed were converted to percentage scores. Being prepared was defined as obtaining a 50% or greater score within the technical areas and overall.

Using data on related capabilities from the GHS Index conducted in 2019, the weighted scores for each of the six categories were used to measure performance in each of the categories. The overall score was used as a measure of each country’s preparedness, where overall score = ∑ category scores, and category score = ∑ weighted indicator scores. The GHS Index scoring system was adopted to rate the countries as having low scores (0.0% – 33.3%), moderate scores (33.4% – 66.6%) or high scores (66.7% – 100.0%). A concordance analysis, using Cohen’s kappa, was used to determine the agreement between the WHO JEE and the GHS Index, with an agreement of 0.61 ≤ κ ≤ 0.80 considered as ‘substantial’.^17^ Kendall’s coefficient of concordance was also calculated for concordance between the ratings using the JEE and GHS Index measurement scales. A Kendall’s coefficient of concordance > 0.5 with an associated *p* < 0.05 was considered a strong agreement.

STATA^®^ version 16 (StataCorp LLC, College Station, Texas, United States) was used for statistical analysis. *p* < 0.05 was considered statistically significant. Student *t*-tests were used to compare the mean number of cases per million population by performance in the JEE categories or GHS indices rating.

## Results

Forty-two (89%) of the 47 member states of the WHO African Region (WHO/AFRO) were included; the other five (11%) had no JEE and GHS Index data. Of the 19 JEE core capacities, only four (21%) categories, including immunisation, laboratory systems, real-time surveillance, and workforce development, had a mean score of at least 50%. The overall performance of the 42 countries in the 19 core capacities was below 50% (mean: 40%; standard deviation [s.d.]: 9) (Table 3).

**TABLE 3:** Performance of the 42 WHO African Region member states in the 19 core capacities of the JEEs conducted between 2016 and 2019.

Summary findings	Legislation	Coordination	AMR	Zoonotic	Food safety	Biosafety	Immunisation	Laboratory	Surveillance	Reporting	Workforce	Preparedness	Emergency response	Public health authorities	Medical countermeasures	Risk communication	Ports of entry	Chemical events	Radiation	Overall JEE (%)
Mean	37	38	29	46	39	33	67	50	57	45	50	29	37	40	27	46	29	32	30	40
Standard deviation	14	17	12	17	17	12	13	15	12	8	13	9	15	22	13	14	15	14	12	9
Minimum	20	20	20	13	20	20	30	20	25	30	20	20	20	20	20	24	20	20	20	26
Maximum	80	80	70	80	100	60	100	75	80	60	73	50	75	100	80	76	80	80	60	62

AMR, antimicrobial resistance; JEE, Joint External Evaluation.

In the GHS Index evaluation, the overall performance of the 42 countries was below 50% in all the six categories of prevention, detection and reporting, rapid response, status of health systems, compliance with international norms and standards for biosafety and biosecurity, and risk and vulnerability of the country system (Table 4). The overall performance was also low (mean: 32; s.d.: 7.1).

**TABLE 4:** Performance of the 42 WHO African Region member states in the GHS Index evaluation conducted in 2019.

Summary findings	Prevention	Detection and reporting	Rapid response	Health systems	Compliance with norms	Risk environment	Overall score
Mean (%)	27	35.0	32.0	15.0	48.0	40.0	32.0
Standard deviation	9	16.0	11.0	7.0	10.0	12.0	7.1
Minimum	10	6.1	16.0	4.6	29.1	20.1	20.0
Maximum	46	82.0	58.0	33.0	72.0	71.0	50.0

Using the JEE-based ratings (not prepared: 0.0% – 49.0%, prepared: 50.0% – 100.0%), overall, the 42 countries were rated as ‘prepared’ in only five of the 19 technical areas, including immunisation (95.0%), real-time surveillance (81.0%), laboratory systems (62.0%), workforce development (60.0%), and reporting (55.0%). Using the GHS Index scoring system (low score: 0.0% – 33.3%; moderate score: 33.4% – 66.6%; high score: 66.7% – 100.0%), ≥ 50.0% of the countries had medium scores in three categories, including detection and reporting (*n* = 21; 50%), risk environment (*n* = 29; 70.0%), and compliance with international norms (*n* = 38; 90.0%). Few member states had high scores, and these were in the risk environment (*n* = 1; 3.0%), compliance to international norms (*n* = 2; 5.0%), and detection and reporting (*n* = 2; 5.0%) categories. Notably, no country had a low score for compliance with international norms; all had either high (*n* = 2) or moderate (*n* = 40) scores. There was a strong overall agreement between the JEE and GHS Index percentages (Kendall’s coefficient of concordance = 0.7; *p* = 0.003).

The rate of increase in the number of COVID-19 cases from Day 1 to Day 90 was 14.3 cases per million people per week (*n* = 41, s.d.: 21.1, range: 0.6–114.0) and from Day 90 to Day 120 was 49.8 cases per million population per week (*n* = 42, s.d.: 106.8, range: 98.6–577.1).

**FIGURE 1:**
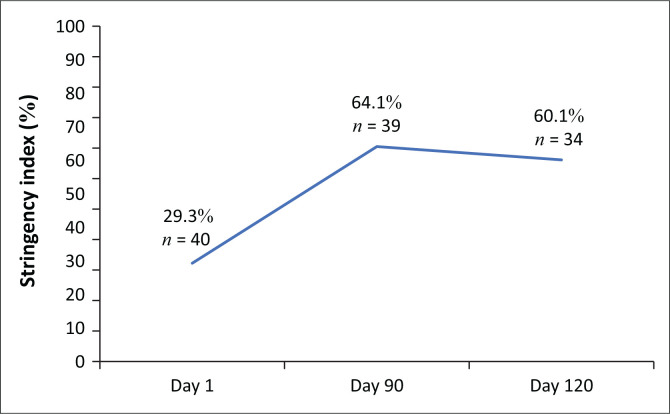
Average stringency index of 42 WHO African Region countries at Day 1 (28 February 2020 – 15 May 2020); and Day 120 (120 days from the date of the first reported case; range: 26 June 2020 – 11 September 2020).

The stringency index, calculated as the mean score of the response indicators of school closures, workplace closures, public events, use of public transport, lockdowns, and internal and international travel, was evaluated. Each indicator, taking a value between 0 and 100 (100 = strictest), increased from an average of 29.3% at Day 1 to 64.1% by Day 90, and slightly reduced to 60.1% by Day 120 (Figure 1).

There were no significant differences in reported cases per million people on Day 1, Day 90, and Day 120 by performance rating in all the GHS Index categories (Table 5). At Day 1, the mean number of COVID-19 cases per million population was significantly higher among countries rated as ‘prepared’ based on overall JEE scores compared to countries rated as ‘not prepared’ (*p* = 0.01) (Table 6). The mean number of COVID-19 cases per million population at Day 1 was also significantly higher in the countries rated as ‘prepared’ in the following JEE core capacities: legislation, policy and finance (3.0 vs 0.2; *p* = 0.03), international health regulation coordination, communication and advocacy (2.9 vs 0.3; *p* = 0.03), food safety (3.4 vs 0.2; *p* = 0.01), and ports of entry (4.8 vs 0.2; *p* = 0.001). Conversely, at Day 90, the mean number of COVID-19 cases per million population was significantly higher in the countries rated as ‘not prepared’ in the core competencies of national laboratory systems (298.3 vs 121.5; *p* = 0.05) and availability of real-time surveillance (376.8 vs 145.3; *p* = 0.04). However, the mean number of COVID-19 cases per million population was still significantly higher in the countries rated as ‘prepared’ in the JEE immunisation category compared to the countries rated as ‘not prepared’ (1589.8 vs 579.4; *p* = 0.01). At Day 120, the mean number of COVID-19 cases per million population was significantly higher in the ‘not prepared’ countries in the JEE immunisation category (1370.2 vs 370.3; *p* = 0.04) but higher in the ‘prepared’ countries in the JEE preparedness (1167.0 vs 359.7; *p* = 0.04) and ports of entry (1046.1 vs 330.9; *p* = 0.02) categories.

**TABLE 5:** Mean number of cases per million population and the performance of the 42 WHO African Region countries in the GHS Index evaluations conducted in 2019.

Rating in each GHS Index category	Mean number of cases/million population											
	Day 1				Day 90				Day 120			
	*n*	Mean	s.d.	*p* *	*n*	Mean	s.d.	*p* *	*n*	Mean	s.d.	*p* *
**Prevention**												
Low	35	1.0	3.4	0.6	34	193.9	286.9	0.9	33	367.9	515.1	0.3
Moderate	7	0.1	0.3	-	7	173.9	263.6	-	7	667.0	1172.0	0.3
**Response**												
Low	28	1.0	3.8	0.5	27	208.5	316.6	0.6	26	423.4	578.7	0.1
Moderate	14	0.3	0.6	-	14	155.6	196.7	-	14	414.4	826.8	-
**Health systems**												
Low	42	0.8	3.1	-	41	190.5	279.8	-	40	420.3	665.3	-
Moderate	0	-	-	-	0	-	-	-	0	-	-	-
**Compliance**												
High	2	0.1	0.1	0.7	2	72.9	21.7	0.5	2	189.1	18.9	0.6
Moderate	40	0.8	3.2	-	39	196.5	285.9	-	38	432.4	680.8	-
**Detection and reporting**												
Low	28	1.0	0.4	0.4	18	247.2	378.4	0.3	17	438.2	675.8	0.9
Moderate	14	0.3	4.2	-	23	146.1	165.0	-	23	407.0	672.4	-
**Risk environment**												
Low	2	1.2	1.6	0.8	2	223.4	58.8	0.8	1	269.7	-	-
Moderate	40	0.7	3.2	-	39	188.8	287.0	-	39	424.1	673.6	-
**GHS Index overall**												
Low	24	1.1	0.8	0.4	23	224.5	71.1	0.4	22	453.8	135.4	0.7
Moderate	18	0.2	0.1	-	18	147.0	41.2	-	18	379.3	169.1	-

s.d., standard deviation; GHS, Global Health Security.

* , Comparison of the mean number of cases by GHS Index rating (low vs moderate) using the Student’s *t*-test.

**TABLE 6:** Mean number of cases per million population and performance of the 42 WHO African Region countries in the JEE conducted between 2016 and 2019.

Joint External Evaluation categories	Mean number of cases per million population											
	Day 1				Day 90				Day 120			
	*n*	Mean	s.d.	*p* *	*n*	Mean	s.d.	*p* *	*n*	Mean	s.d.	*p* *
**Overall JEE**												
**Overall**												
Not Prepared	35	0.3	0.3	0.01	34	194.4	290.0	0.08	33	363.4	523.5	0.02
Prepared	7	3.3	7.6	-	7	171.2	243.6	-	7	688.2	1146.1	-
**Prevention**												
**Legislation, policy and finance**												
Not Prepared	34	0.2	0.3	0.03	33	220.6	302.6	0.02	32	465.4	722.3	0.04
Prepared	8	3.0	7.1	-	8	66.4	87.2	-	8	239.9	339.7	-
**International Health Regulation coordination, communication and advocacy**												
Not Prepared	34	0.3	0.3	0.03	33	188.1	294.6	0.09	32	354.8	534.3	0.02
Prepared	8	2.9	7.1	-	8	200.5	226.3	-	8	682.1	1050.7	-
**Antimicrobial resistance**												
Not Prepared	40	0.8	3.2	0.06	39	197.9	285.1	0.04	38	438.5	677.9	0.05
Prepared	2	0.1	0.1	-	2	45.7	41.7	-	2	73.7	71.4	-
**Zoonotic**												
Not Prepared	22	1.1	4.3	0.04	22	220.6	346.6	0.05	21	373.8	620.2	0.06
Prepared	20	0.4	0.6	-	19	155.6	177.8	-	19	471.6	725.5	-
**Food safety**												
Not Prepared	37	0.2	0.1	0.01	34	194.6	290.0	0.08	33	356.6	526.4	0.02
Prepared	5	3.4	2.8	-	7	170.6	244.2	-	7	720.4	1127.8	-
**Biosafety and biosecurity**												
Not Prepared	37	0.9	3.3	0.06	36	194.5	282.0	0.08	35	381.0	519.1	0.03
Prepared	5	0.03	0.03	-	5	161.6	294.8	-	5	695.4	1384.6	-
**Immunisation**												
Not Prepared	3	0.3	0.2	0.08	3	579.4	814.5	0.01	2	1370.2	1837.8	0.04
Prepared	39	0.8	3.2	-	38	1589.8	188.8	-	38	370.3	569.2	-
**Detection**												
**National Laboratory Systems**												
Not Prepared	16	0.3	0.3	0.04	16	298.3	385.3	0.05	15	526.9	695.8	-
Prepared	26	1.1	4.0	-	25	121.5	158.6	-	25	356.3	652.3	0.04
**Real-time surveillance**												
Not Prepared	8	0.3	0.3	0.06	8	376.8	534.0	0.04	8	657.1	879.8	-
Prepared	34	0.9	3.5	-	33	145.3	157.6	-	32	361.1	587.3	0.03
**Reporting**												
Not Prepared	19	0.4	0.4	0.05	18	167.3	215.6	0.06	18	277.8	361.7	0.02
Prepared	23	1.1	4.3	-	23	208.6	325.3	-	22	536.8	827.3	-
**Workforce development**												
Not Prepared	17	0.3	0.4	0.05	16	260.4	391.8	0.02	15	514.4	697.6	0.05
Prepared	25	1.0	4.1	-	25	145.7	171.3	-	25	363.8	653.1	-
**Response**												
**Preparedness**												
Not Prepared	39	0.8	3.2	1.00	38	179.9	277.5	0.04	37	359.7	511.5	0.04
Prepared	3	0.8	1.4	-	3	324.9	337.0	-	3	1167.0	1737.3	-
**Emergency operation centres**												
Not Prepared	34	0.9	3.5	0.07	33	189.7	295.1	1.00	32	385.2	543.5	0.05
Prepared	8	0.4	0.8	-	8	193.7	223.4	-	8	560.4	1063.0	-
**Linking public health and security**												
Not Prepared	33	0.8	3.5	0.08	33	194.0	295.2	0.09	32	383.9	546.5	0.05
Prepared	9	0.5	0.8	-	8	176.1	221.7	-	8	565.8	1055.1	-
**Risk communication**												
Not Prepared	30	0.3	0.4	0.01	29	214.1	309.7	0.04	28	404.4	569.0	0.08
Prepared	12	2.0	5.8	-	12	113.5	189.5	-	12	457.2	894.6	-
**Other**												
**Ports of entry**												
Not Prepared	37	0.2	0.3	0.001	36	176.6	284.4	0.04	35	330.9	515.5	0.02
Prepared	5	4.8	8.7	-	5	290.2	249.3	-	5	1046.1	1223.9	-

s.d., standard deviation; JEE, Joint External Evaluation.

* , Comparison of the mean number of cases by JEE rating (prepared vs not prepared) using the Student’s *t*-test.

## Discussion

At the onset of the pandemic in WHO/AFRO member countries on 14 February 2020, 44 countries had conducted and published their JEE reports as an indicator of preparedness of their systems for any event of public health concern. Although the 42 countries considered in this review scored below 50% on average, indicating a lack of preparedness, there were notable areas where systems were in place. These included structures for immunisation, laboratory systems, surveillance, and workforce development. Over the years, many African countries have conducted and built systems for immunisation of children under five years, leading to successes like the elimination of the wild poliovirus.^18^ In addition, numerous outbreaks of cholera, Ebola, influenza, Rift Valley fever and other endemic diseases like malaria may have contributed to the observed existence of surveillance systems. Since 2009, WHO/AFRO, in collaboration with the Africa Society for Laboratory Medicine and other partners, has developed and implemented the Strengthening Laboratory Management Towards Accreditation training programme and the Strengthening Laboratory Quality Improvement Process Towards Accreditation programme across Africa.^19,20^ This may have contributed to the preparedness of the laboratory systems as reported by the JEE.

Nevertheless, WHO/AFRO countries were generally unprepared for a global pandemic as determined by both the WHO JEE and GHS Index assessments. There was a lack of preparedness in key core capacities such as legislation, coordination, preparedness, emergency response, medical countermeasures, risk communication, and ports of entry. In the early phases of the epidemic, all reported cases were imported through the various ports of entry. Most countries did not have legislation to empower governments to institute some of the public health measures needed to control the epidemic, resulting in delays and court challenges in some instances.^21^ It took time to mobilise all the coordination mechanisms required, especially the activation of the emergency operation centres and the establishment of COVID-19 task teams, resulting in disaggregated responses in the early days. Although the concept of a rapid response team was known and, in some cases, documented, these had not been tested at the scale needed and the anxiety associated with the disease delayed the full activation and deployment of the rapid response teams.

There are two additional areas measured by the GHS Index that provide additional measures of preparedness. These include the country’s vulnerability to biological threats and the overall risk environment, as well as the sufficiency and robustness of health systems to treat the sick and protect health workers. The GHS Index suggests that health systems in WHO/AFRO countries are underdeveloped, with the 42 countries having their lowest scores in the health systems category among the six indicators of preparedness. Deficiencies identified in the health systems by the GHS Index include poor infrastructure, lack of dedicated finance from fiscus, and lack of documented commitment to prioritising healthcare services for healthcare workers who participate in a public health response, among others.^15^ The GHS Index also showed that WHO/AFRO did not have a conducive environment in terms of political systems and government effectiveness in dealing with epidemics, as indicated by the low score in the risk environment category.^15^ A sizeable number of countries in the region have political and security risks, including civil wars, political and economic instabilities, and other cross-boundary disputes that could undermine national capabilities to counter threats. The Ebola epidemic in West Africa was an example where accessibility to affected areas was hampered by civil strife.^22^

Did the level of readiness of WHO/AFRO member countries as depicted by the WHO JEE and GHS Index have a bearing on how the epidemic spread to and within Africa? The authors examined the onset of the epidemic and its progression from Day 1, the date of the first reported case in each country, to Day 120. This was done because, after 120 days, the status or progression of the epidemic may have been affected by the rapid changes adopted by the countries following the realisation of its possible social and economic impacts.

Preparedness, as measured by the GHS Index indicators, was found to have no significant impact on the mean number of cases per million population from the onset of the epidemic until Day 120. However, at the onset of the pandemic (Day 1), the mean number of cases was statistically significantly higher among countries rated as ‘prepared’ compared to countries rated as ‘not prepared’ based on overall JEE scores and scores in the food safety, ports of entry, legislation, policy and finance, and international health regulation JEE core capacities. With an average overall JEE score of 40%, the poor status of overall preparedness of countries in the region seems to have had an impact on the onset and spread of the disease in the region. Coordination, legislation, and advocacy are key to mounting a response in the event of a threat like the COVID-19 pandemic. All initial cases in the region were imported through the various ports of entry, which, besides the airports, are largely very porous. The designated ports of entry were not adequately prepared, with no designated screening and isolation infrastructure or adequate and appropriately trained personnel resources to deal with COVID-19.^23^ This calls for the strengthening of early warning systems similar to the WHO Global Influenza Surveillance and Response System.^24^

As the epidemic progressed, countries began to mobilise resources and set coordination structures, including the activation of emergency operation centres and the establishment of COVID-19 coordination mechanisms like task force teams that were reporting to the highest offices in the country. Implementation of public health measures, including lockdowns, was the most immediate response for most countries. This is reflected in the reported increase in stringency index from 29.3% at the onset to 64.1% by Day 90. Early in the pandemic, the WHO established the identification of cases through laboratory testing as central to the response.^25^ Consequently, most countries prioritised localising diagnostic capacity and, with support from the WHO and the Africa Centres for Disease Control and Prevention, the number of countries able to confirm COVID-19 increased from 2, when the first case was reported in Africa on 14 February 2020, to 24 countries able to confirm severe acute respiratory syndrome coronavirus 2 (SARS-CoV-2) infection by 20 February 2020.^26^ This supports the observed higher rating of the laboratory system capacity in most countries 120 days into the pandemic.

### Limitations

The findings of the study may be limited by inconsistent reporting in the early days of the pandemic as countries were establishing various capacities. Through external funding, many countries made rapid changes within the first few months of detecting the first cases. This may have altered the relationship between baseline preparedness rating and the mean number of cases at Day 90 and Day 120 after the first case. We did not have enough data to adjust for these rapid interventions. Despite the weaknesses of the data collection tools, the data generated using the JEE and GHS Index was critical in drawing international attention to the critical areas for building the capacity of nations to prevent, detect, and respond to epidemic threats.

### Conclusion

This analysis highlights the importance of building capacity for pandemic preparedness and response at multiple levels. At the onset of the COVID-19 pandemic in February 2020, WHO/AFRO member countries were not adequately prepared as measured by the WHO JEE and the GHS Index. Countries’ ratings in the legislation, policy and finance, and IHR coordination, communication and advocacy WHO JEE categories, as well as in the health systems GHS Index category, were not optimal. Given all the lessons learnt during the COVID-19 pandemic, including the rapid global spread and emergence of variants of concern, critical areas that predict the successful handling of epidemics need to be assessed.
